# Engineering *Bacillus thuringiensis* Cyt1Aa toxin specificity from dipteran to lepidopteran toxicity

**DOI:** 10.1038/s41598-018-22740-9

**Published:** 2018-03-21

**Authors:** Mary-Carmen Torres-Quintero, Isabel Gómez, Sabino Pacheco, Jorge Sánchez, Humberto Flores, Joel Osuna, Gretel Mendoza, Mario Soberón, Alejandra Bravo

**Affiliations:** Departamento de Microbiología, Instituto de Biotecnología, Universidad Nacional Autónoma de México, Apdo. postal 510-3, Cuernavaca, 62250 Morelos Mexico

## Abstract

The Cyt and Cry toxins are different pore-forming proteins produced by *Bacillus thuringiensis* bacteria, and used in insect-pests control. Cry-toxins have a complex mechanism involving interaction with several proteins in the insect gut such as aminopeptidase N (APN), alkaline phosphatase (ALP) and cadherin (CAD). It was shown that the loop regions of domain II of Cry toxins participate in receptor binding. Cyt-toxins are dipteran specific and interact with membrane lipids. We show that Cry1Ab domain II loop3 is involved in binding to APN, ALP and CAD receptors since point mutation Cry1Ab-G439D affected binding to these proteins. We hypothesized that construction of Cyt1A-hybrid proteins providing a binding site that recognizes gut proteins in lepidopteran larvae could result in improved Cyt1Aa toxin toward lepidopteran larvae. We constructed hybrid Cyt1Aa-loop3 proteins with increased binding interaction to *Manduca sexta* receptors and increased toxicity against two Lepidopteran pests, *M. sexta* and *Plutella xylostella*. The hybrid Cyt1Aa-loop3 proteins were severely affected in mosquitocidal activity and showed partial hemolytic activity but retained their capacity to synergize Cry11Aa toxicity against mosquitos. Our data show that insect specificity of Cyt1Aa toxin can be modified by introduction of loop regions from another non-related toxin with different insect specificity.

## Introduction

During sporulation, *Bacillus thuringiensis* (Bt) produce different kinds of insecticidal toxins such as Cry and Cyt toxins that have been used commercially worldwide to control different insect pests^1,2^. The family of Cry toxins composed of three-domains (3d-Cry) and the Cyt toxins of Bt are not related phylogenetically. Different 3d-Cry toxins show high insecticidal activity against dipteran, lepidopteran and coleopteran larvae or against nematodes. In contrast, the Cyt toxins show mainly dipteran specificity^1,3^. However, Cyt toxins also display relatively low toxicity against other insect orders such as coleopteran, hemipteran and hymenopteran suggesting that Cyt toxins could be potentially useful for the control of insect pest different from mosquitoes^4–10^. It is interesting that Cyt toxins have been frequently found in Bt strains that express dipteran specific 3d-Cry toxins and that Cyt1A is able to synergize the activity of Cry4Aa, Cry4Ba or Cry11Aa toxins against mosquitoes^11,12^. In addition, it was found that Cyt1Aa toxin is able to overcome the resistance of the *Culex quinquiefasciatus* populations to Cry4Aa, Cry4Ba or Cry11Aa toxins^13^.

The Cyt and 3d-Cry toxins are both pore-forming proteins. The Cyt toxins are composed by a single α–β domain^14^ that directly interacts with the membrane lipids, no protein receptor has been described. Cyt toxin insert into the membrane forming pores or by damage the membrane structure by a detergent like interaction^15,16^. On the other hand, the 3d-Cry toxins have a three domain structure, where domain I is involved in pore formation and domains II and III participate in the interaction with different protein receptors, thus conferring specificity to the toxin. The 3d-Cry toxins showed a complex mechanism of action that requires the interaction with several proteins in the insect gut such as aminopeptidase N (APN), alkaline phosphatase (ALP) and cadherin (CAD) before insertion into the membrane and pore formation^1^. The Cry1Ab toxin regions involved in these interactions have been mapped. Domain II loop 3 of Cry1Ab plays an important role in the binding of this toxin to *Manduca sexta* APN and CAD receptors^17^, while loop 2 and loop α-8 also participate in CAD binding^18^. Mutations in these loops have resulted in proteins with lower or increased toxicity against specific insect pests, correlating with lower or higher binding affinities to brush border membrane vesicles (BBMV) from those insects^19^. Modification of loop sequences have resulted in proteins with changed specificity such as the Cry1Aa-hybrid toxin that display similar amino acid sequence to the loops of Cry4Ba toxin, and acquired toxicity to dipteran larvae^20^. Similarly, the introduction of two peptide sequences, selected to bind to BBMV from the hemipteran insect *Nivalopavata lugens*, into the Cry1Ab domain II exposed loops resulted in a Cry1Ab-hybrid toxin with enhanced toxicity against *N. lugens*^21^. In the case of Cyt toxin, construction of hybrid toxins have also resulted in improved toxicity against specific targets, such as the Cyt2Aa-hybrid toxins containing the sequence of a 12 amino acid GBP3 peptide that binds to an aphid APN. These Cyt2A-hybrid toxins were constructed in three different exposed loops of the toxin, showing enhanced toxicity against the hemipteran insects such as *Acyrthosiphon pisum* and *Myzus persicae*^22^.

Here, we describe the rational migration of Cyt1Aa toxicity from dipteran to lepidopteran insects by constructing Cyt1Aa-hybrid toxins containing the sequence of Cry1Ab domain II loop 3. The hypothesis behind the construction of these hybrid toxins is that providing a binding site that recognizes gut proteins in lepidopteran larvae would result Cyt1Aa-hybrid proteins that display toxicity against lepidopteran. We show here that loop 3 of Cry1Ab is involved in the binding interaction with different receptors such as APN, ALP and CAD. We constructed Cyt1Aa-loop3 hybrid proteins containing this loop 3 sequence in different locations of Cyt1Aa. Some of these Cyt1Aa-hybrid proteins showed increased binding to *M. sexta* receptors that correlated with insecticidal activity against two lepidopteran species, *M. sexta* and *Plutella xylostella*. Our data shows that insect specificity of Cyt toxins can be modified by the introduction of loop regions from non-related toxins with different insect specificity.

## Results

### Binding analysis of Cry1Ab-G439D mutant protein to APN, ALP and CAD receptors

The cloning of the recombinant ALP and CAD fragment containing Cry1Ab binding sites (CAD-CR12) was previously reported^17,23^. Here we cloned the APN1 gene from *M. sexta* and expressed it into *E. coli* cells as reported in Materials and Methods.

**Figure 1:**
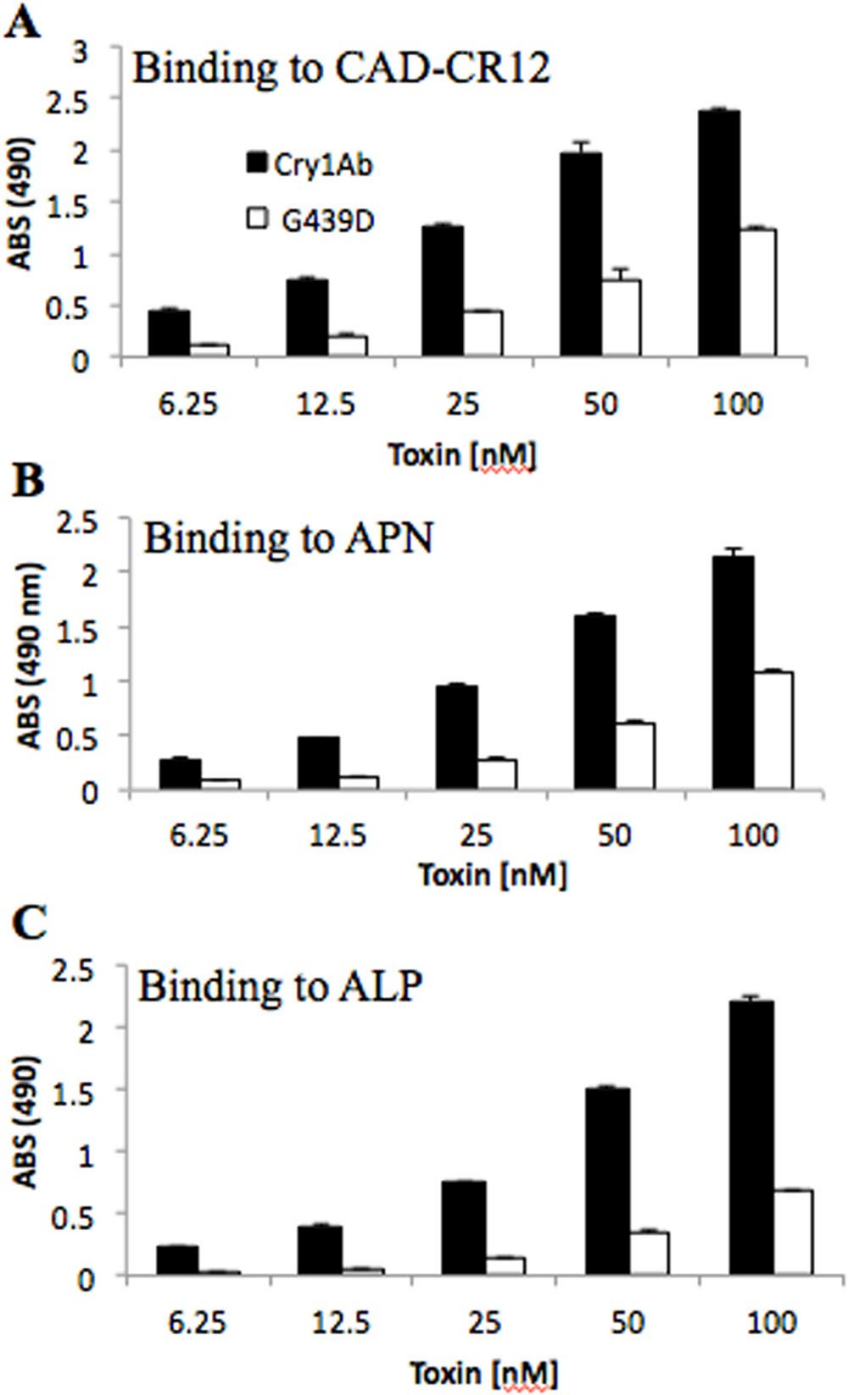
Analysis of binding of Cry1Ab-G439D mutant toxin in loop3 of domain II to purified APN1, ALP or CAD proteins. ELISA plates were coated with 0.5 μg of each recombinant receptor protein (APN1, ALP or CAD) and the binding of different concentrations of wild type or mutant toxin was analyzed. Each experiment was performed in duplicate with a total of six repetitions for each toxin.

It was previously reported that mutants in loop 3 region from Cry1Ab are affected in toxicity to *M. sexta* and showed reduced binding to APN1 and CAD receptors as compared with the wild type Cry1Ab toxin^17^. Also, that Cry1Ab-G439D loop 3 mutant was affected in binding to CAD-CR12^24^. The binding of Cry1Ab loop3 mutants to ALP receptor was not analyzed before. Here we analyzed the binding of Cry1Ab-G439D loop 3 mutant to ALP, APN1 and CAD-CR12. Figure 1 shows that Cry1Ab-G439D mutant was also affected in binding to ALP, indicating that Cry1Ab domain II loop 3 is also involved in the binding with this receptor.

### Construction of Cyt1Aa-loop3 hybrid toxins

**Figure 2:**
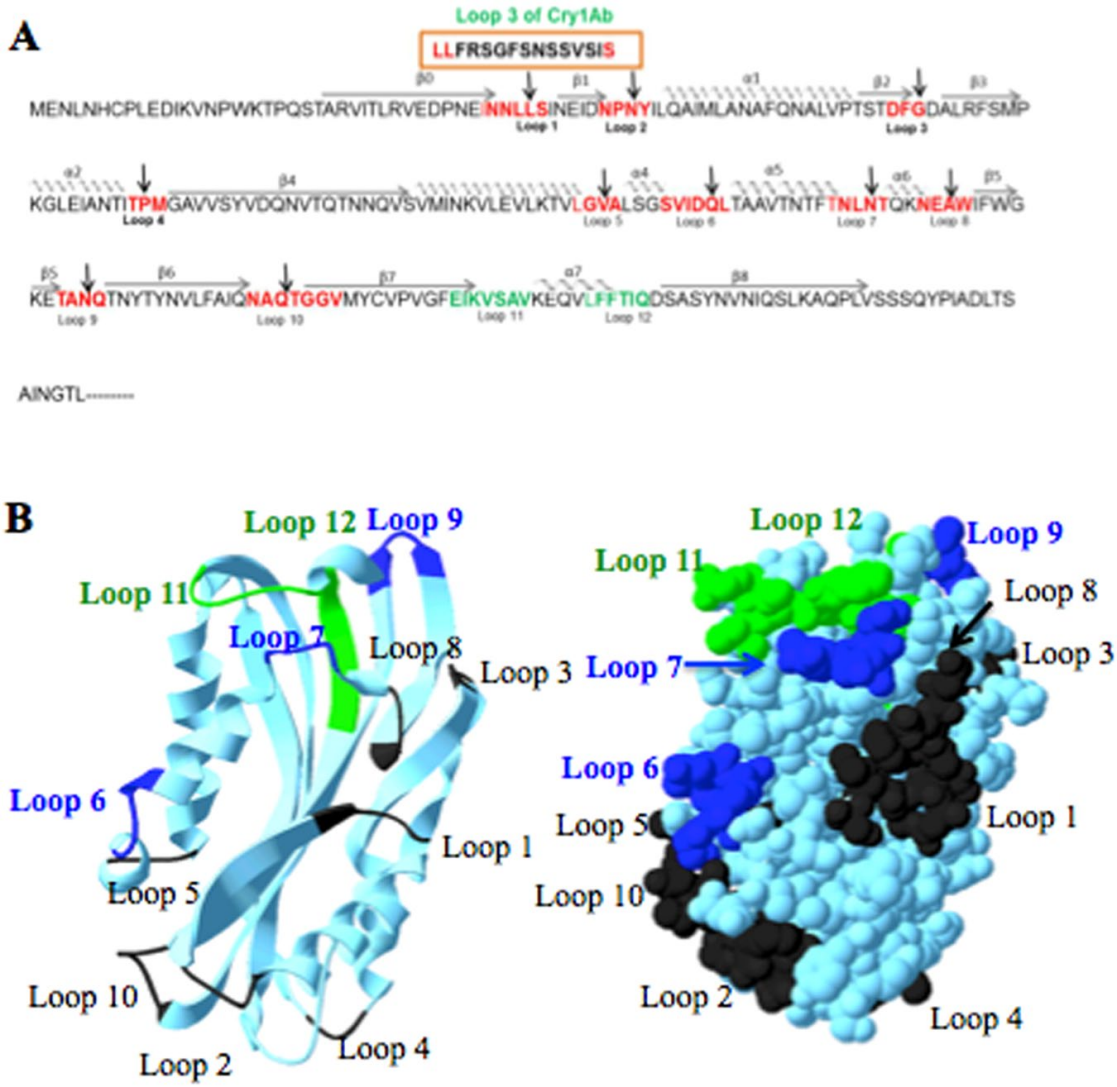
Location of loops regions in Cyt1Aa toxin where loop3 from Cry1Ab was inserted. Panel A, Amino acid sequence of Cyt1Aa toxin is aligned with the three-dimensional structures, helices alpha and beta strands are labeled over the amino acid sequence. The selected loop regions are labeled in red, arrows indicate the site of insertion of loop3 (FRSGFSNSSVSI) from Cry1Ab. Residues labeled in green correspond to regions involved in synergism with Cry11Aa and Cry4Ba. Panel B, Localization of loops in the three dimensional structure of Cyt1Aa (PDB 3RON) showing that the selected regions were exposed to the solvent. Loop regions that resulted in improved toxicity against lepidopteran larvae are in blue dark color. Loop regions important for synergism are in green color.

Based in the three-dimensional structure of Cyt1Aa toxin^25^ we identified twelve exposed loop regions. We selected ten of these loops to insert the amino acid sequence of the Cry1Ab domain II loop3 (FRSGFSNSSVSI) (Fig. 2). We did not insert the peptide sequence into loops 11 and 12 of Cyt1Aa since it was previously shown that these loops were involved in the binding interaction and synergism with Cry11Aa and Cry4Ba toxins^11,12^. The loop regions of Cyt1Aa that where selected to introduce the Cry1Ab loop3 are highlighted with red letters in Fig. 2A. The location of these loops in the Cyt1Aa structure showed that all of them are exposed to the solvent (Fig. 2B).

**Figure 3:**
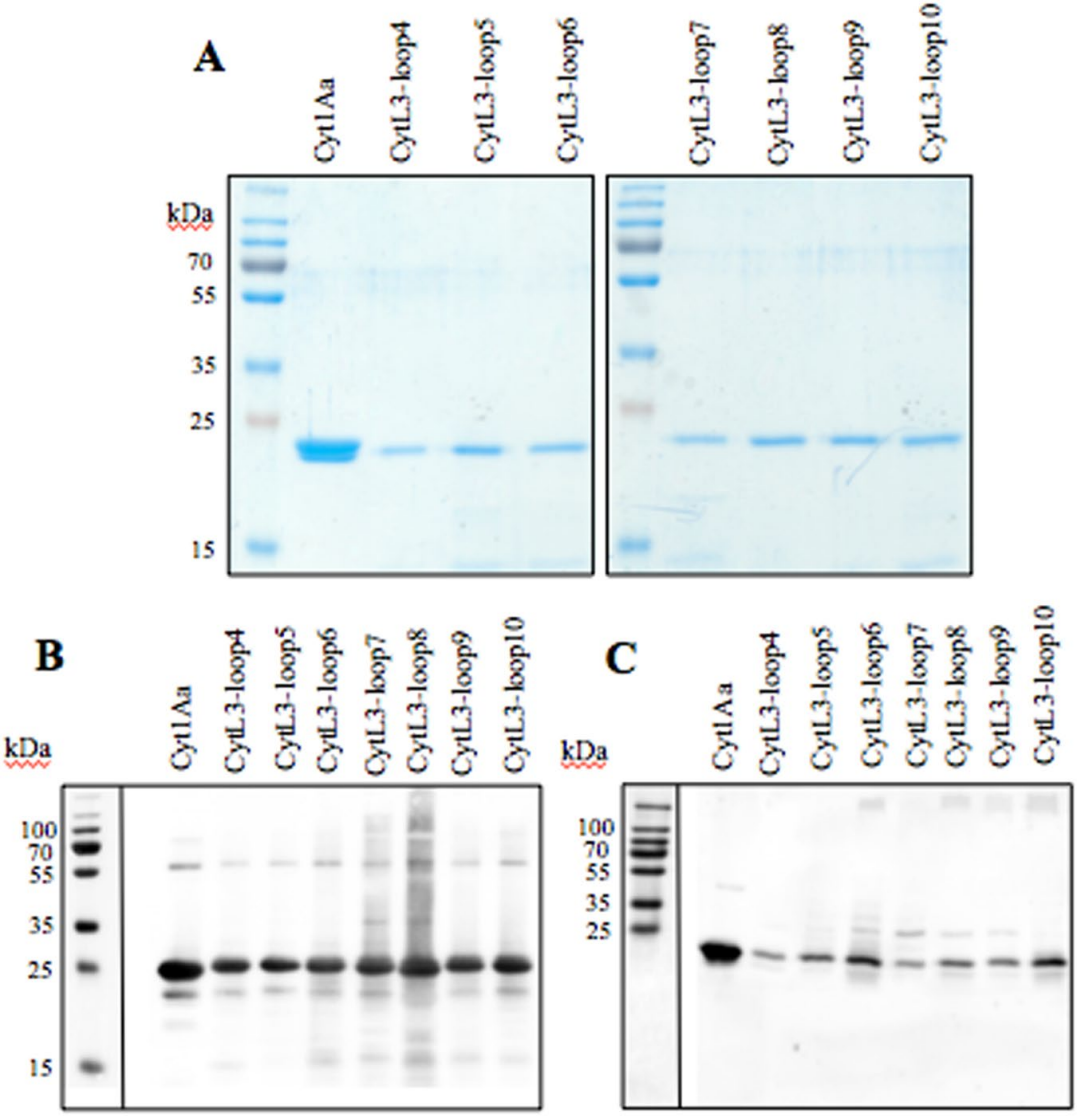
Purification of Cyt1Aa-hybrid proteins expressed in *B. thuringiensis* 407^-^ strain. Panel A, purified trypsin activated toxins resolved in SDS-PAGE 15% acrylamide stained with Coomassie blue. Panel B, analysis of hybrid Cyt1Aa protoxins by western blot assays using anti-Cyt1Aa polyclonal specific antibody. Panel C, analysis of hybrid Cyt1Aa activated toxins by western blot assays using anti-Cyt1Aa polyclonal specific antibody. Page ruler plus pre-stained protein ladder in panels B and C are shown as separate images.

Ten hybrid-Cyt1Aa mutants were constructed and confirmed by DNA sequence. The presence of crystal-like inclusions was confirmed by observations under phase contrast optical microscopy and by SDS-PAGE gels of protoxins. SDS-PAGE gels showed that constructs in loop1, loop2 and loop3 of Cyt1Aa did not produce protein in Bt and were not further analyzed (data not shown). The crystal-like inclusions from the remaining seven constructs were purified and the protoxins were solubilized and activated with trypsin. Figure 3A shows the purified trypsin activated toxins, as well as the western blot detection of the solubilized (Fig. 3B) and activated proteins (Fig. 3C).

### Binding analysis of Cyt1Aa-hybrid proteins to APN, ALP and CAD receptors

**Figure 4:**
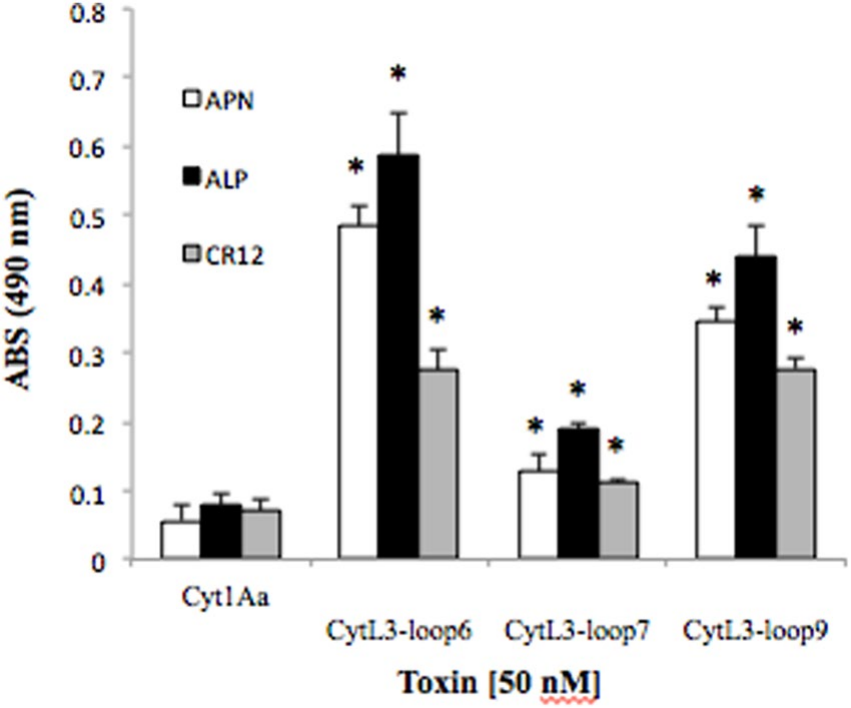
Analysis of binding of Cyt1Aa and hybrid-Cyt1Aa variants to purified APN1, ALP or CAD proteins. ELISA plates were coated with 0.5 μg of each recombinant receptor protein (APN1, ALP or CAD) and the binding of 100 nM Cyt1Aa or hybrid-Cyt1Aa was analyzed. Each experiment was performed in duplicate with a total of six repetitions for each mutant Cyt1Aa toxin. *Indicate significant differences with the Cyt1Aa toxin by ANOVA with significant differences P < 0.01.

The binding of Cyt1Aa-hybrid variants to recombinant *M. sexta* APN1, ALP and CAD-CR12 was analyzed by ELISA binding assays. The variants CytL3-Loop6, CytL3-Loop7 and CytL3-Loop9 showed significant binding to the three receptor proteins (Fig. 4), showing 2 to7 fold higher binding to ALP; 2 to 8 fold higher binding to APN1, and 1.5 to 4 fold higher binding to CAD-CR12 when compared with the binding of the control Cyt1Aa toxin (ANOVA analysis P < 0.005). The rest of the hybrid Cyt1A proteins showed less binding to the *M. sexta* receptors than CytL3-Loop7 (data not shown).

### Toxicity against mosquitoes and hemolytic activity

The toxicity of the seven hybrid-Cyt1Aa mutants and the Cyt1Aa was analyzed in bioassays by feeding third instar *A. aegypti* larvae with the purified crystal-like suspensions. The medium lethal concentration of wild type Cyt1Aa was 146.2 ng/ml (32–253, 95% confidential limits). In contrast, the hybrid-Cyt1Aa mutants showed no toxicity against *A. aegypti* even at 10 μg/ml (maximal concentration used) (data not shown). The toxicity of these constructions was also tested in bioassays using 10 μg/ml of soluble protein against first instar *A. aegypti* larvae as described in materials and methods. Under these conditions all proteins including the wild type Cyt1Aa were inactive (data not shown).

**Figure 5:**
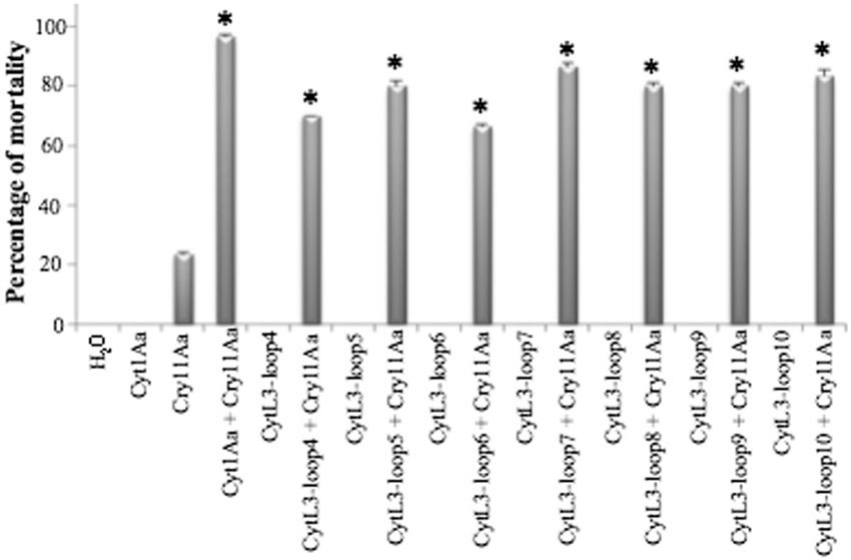
Analysis of synergism of Cyt1Aa or Cyt1Aa-hybrid toxins with Cry11Aa toxin. Cry11Aa toxin was used at 108 ng/ml and Cyt1Aa or hybrid-Cyt1Aa were used at 30 ng/ml. Negative control (dechlorinated water) was included in the bioassay. Larvae mortality was examined 24 h after treatment. *Indicate significant differences with the Cyt1Aa toxin by ANOVA with significant differences P < 0.01.

Cyt1Aa protein synergizes Cry11Aa activity and has hemolytic activity. In order to determine the functionality of these Cyt1Aa-hybrid proteins we analyzed these two activities. The synergism with Cry11Aa toxin was determined by performing bioassays against early *A. aegypti* 3^th^ -instar larvae as described in Materials en Methods. We used a low concentration of these proteins that accounts for extremely low mortality when tested individually. Mortality was 20% with Cry11Aa alone (108 ng/ml Cry11Aa) and no-mortality was observed with Cyt1Aa or hybrid-Cyt1Aa alone (30 ng/ml Cyt1Aa) (Fig. 5). However, when both proteins Cyt1Aa and Cry11Aa were assayed together in the same mixture, more than 95% mortality was observed revealing synergism between these two proteins. The synergism factor (SF) of the wild type proteins was calculated as reported in Materials in Methods showing a SF value of 17.2 fold. Results obtained with the Cyt1A-hybrid proteins showed that mixtures of all of these mutants with Cry11Aa resulted in synergistic activity since mortality increased up to 70 to 90% (Fig. 5). The synergism was also analyzed by testing for deviation from the null hypothesis of simple independent action, which assumes that the proportion of larvae surviving to the exposure of mixture of toxins is the product of the proportions of larvae that survive to the exposure of each toxin separately^26^. The results show that synergism occurred in all mixtures of Cyt1Aa-hybrid toxins with Cry11Aa that were tested (Table 1).

**Table 1:** Synergism between Cry11Aa and Cyt1Aa-hybrid proteins against 3th instar of *Aedes aegypti* larvae.

Toxin	S_(toxin)_ OBS^a^ = (R1 + R2 + R3)/n	S_(mixture)_ EXP^b^ = S_(Cyt1Aa)OBS_ × S_(Cry11Aa)OBS_	Expected mortality^c^ = (1 − S_(mixture)EXP_) × 100%	Observed mortality Cyt1Aa + Cry11Aa^d^
**Cyt1Aa**	1	0.76	24%	97% ± 0.6
**CytL3-Loop4**	1	0.76	24%	70% ± 0
**CytL3-Loop5**	1	0.76	24%	80% ± 1.7
**CytL3-Loop6**	1	0.76	24%	67% ± 0.6
**CytL3-Loop7**	1	0.76	24%	87% ± 1.2
**CytL3-loop8**	1	0.76	24%	80% ± 1.0
**CytL3-Loop9**	1	0.76	24%	80% ± 1.0
**CytL3-Loop10**	1	0.76	24%	83% ± 2.1
**Cry11Aa**	0.76			

^a^Observed survival of individual toxin S(toxin)OBS corresponds to the observed proportion of larvae that survived to the exposure to Cyt1Aa or Cyt1Aa-hybrid mutant toxins. Observed mortality was 20% with Cry11Aa at 108 ng per ml and 0% with Cyt1Aa or mutant toxins at 30 ng per ml. n = 30 larvae for each toxin tested.

^b^Theoretical proportion of larvae that survive to the toxin-mixture, S_(Cyt1Aa, Cry11Aa)EXP_ = S_(Cyt1Aa)OBS_ × S_(Cry11Aa)OBS_ corresponds to the proportion of larvae expected to survive to the exposure of a mixture of toxins.

^c^Theoretical expected mortality was calculated with (1−S_(Cyt1Aa, Cry11Aa)EXP)_ × 100%.

^d^Experimentally observed mortality with the mixture of toxins using Cry11Aa at 108 ng per ml plus each Cyt1Aa-hybrid toxin at 30 ng per ml.

These assays were performed three times. Fisher’s exact test showed values of P < 0.001 for each comparison.

**Figure 6:**
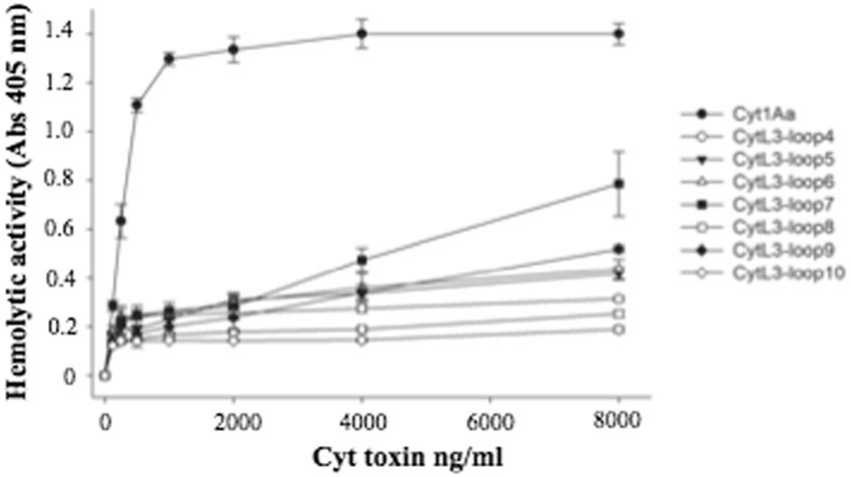
Hemolytic activity of Cyt1Aa and hybrid-Cyt1Aa mutants, Hemolysis was analyzed with rabbit red blood cells. These assays were performed three times and standard deviations are shown.

The hemolytic activity of these constructions was assayed against rabbit red blood cells. The medium effective (EC_50_) dose of Cyt1Aa was 190 ng/mL (182.4–197.8 ng/mL). Figure 6 shows that Cyt1Aa hybrid-mutants are also affected in their hemolytic activity. The maximum concentration of Cyt1A toxin used was 8000 ng per ml of water, at this concentration the hybrid-Cyt1Aa toxins tested showed 1.5 to 7 fold lower hemolytic activity than the wild type Cyt1Aa toxin.

### Toxicity against lepidopteran larvae

**Figure 7:**
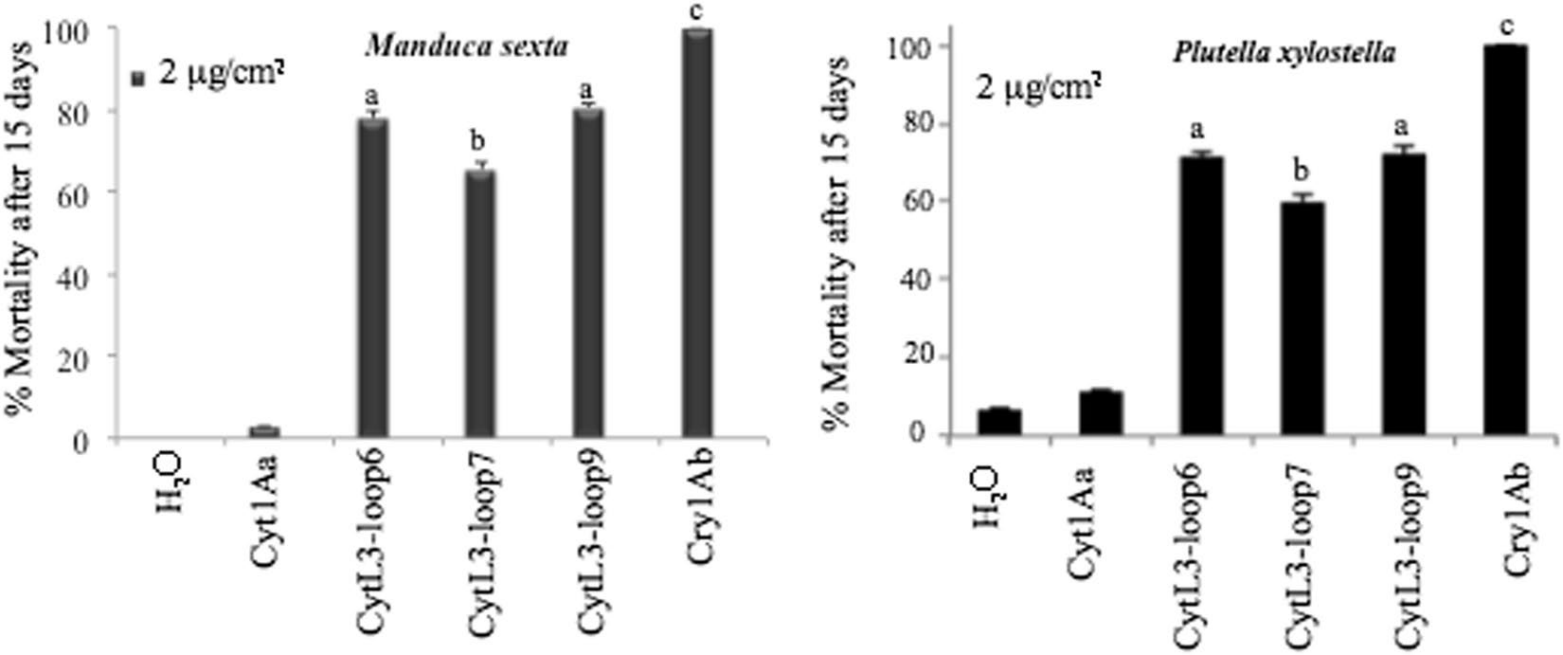
Toxicity of Cyt1Aa or hybrid-Cyt1Aa variants to *Manduca sexta* or *Plutella xylostella* larvae. *M. sexta* neonate or *P. xylostella* third instar larvae were treated with 2 μg of Cyt1Aa or hybrid-Cyt1Aa per cm^2^ artificial diet. The mortality was monitored after 15 days. Twenty-four larvae were used per toxin concentration in triplicate. Letters above the bars indicate significant differences with the Cyt1Aa toxin by ANOVA with significant differences P < 0.01.

Bioassays were performed against two different lepidopteran species, first instar *M. sexta* and third instar *P. xylostella* larvae. After seven days of treatment the larvae of both insects were still alive but showed to be severely affected, since they look pale and showed reduced size in comparison with the water control or the larvae treated with Cyt1Aa (Supplementary Figure S2). After 15 days of exposure to the toxins the mortality was evident (Fig. 7). Anova analysis of the data indicated that constructions CytL3-Loop6, CytL3-Loop7 and CytL3-Loop9 showed significant mortality values that ranged from 60–80% and were significantly more toxic that the other constructions CytL3-Loop4, CytL3-Loop5, CytL3-Loop8 and CytL3-Loop10 and than the control with Cyt1Aa (P < 0.001).

## Discussion

Cyt toxins represent special and interesting proteins since they are toxic to dipteran insects and are able to synergize the insecticidal activity of some 3d-Cry toxins^3^. Cyt1Aa toxin is not toxic to Lepidopteran larvae such as *M. sexta*, it neither synergize toxicity of Cry toxins active against lepidopteran species such as Cry1Ab^11^. Previously, we compared the different steps of Cyt1Aa mode of action in the susceptible dipteran *A. aegypti* with the non-susceptible lepidopteran *M. sexta*^27^. Binding analysis of Cyt1Aa to *M. sexta* BBMV showed 30–50% less binding sites in comparison to *A. aegypti* BBMV, supporting that Cyt1Aa is not toxic to *M. sexta* in part due to reduced number of binding sites^26^.

We constructed hybrid-Cyt1Aa mutants expressing the loop3 of Cry1Ab-domain II in different exposed regions of the Cyt1Aa toxin. The Cyt1Aa-hybrid variants CytL3-Loop6, CytL3-Loop7 and CytL3-Loop9 showed significant binding to APN1, ALP and CAD when compared with the control Cyt1Aa toxin and these constructions showed significant toxicity to two different lepidopteran larvae, *M. sexta* and *P. xylostella*. These data could indicate that the hybrid-Cyt1Aa toxins may be increasing the binding sites of Cyt1Aa *in vivo*, this remains to be analyzed. However, mortality was clearly observed only after 15 days of exposure to the toxins, which differs from Cry1A toxins, which mortality in bioassays is observed after 7 days of exposure to toxin. This indicates that hybrid Cyt1Aa mutants have a distinct and somehow slower mode of action than Cry1A toxins. The Cry toxins have been described as pore forming toxins that induce cell death by forming pores in the membrane of midgut epithelial cells. However, an alternative model proposed that insect death is triggered by the activation of a cascade signal pathway through toxin interaction with CAD receptor. Thus it is also possible that the Cyt1Aa-hybrid variants might be triggering an intracellular pathway during intoxication of the lepidopteran larvae.

Previously, hybrid-mutants were constructed in Cyt2Aa toxin by replacement or by introduction of the GBP3 peptide in different exposed loop regions, resulting in proteins that were able to kill aphid pests^22^. Cyt1Aa and Cyt2Aa shared 35% amino acid identity, and similar three-dimensional structures. Supplementary Figure S3 show a superposition of both structures. In these structures the location of the loops that were selected for insertion of the different peptides are highlighted. The structure of loop1 was not accurately resolved in the three-dimensional structure of Cyt1Aa toxin and the construction of the hybrid mutant in this loop1 resulted in an unstable protein. In the case of the hybrid–Cyt2Aa mutants, GBP3 constructs in loop1, loop3 and loop4 retained insecticidal activity against *A. aegypti* larvae, increased binding to membranes from the aphid insects *A. pisum* and *M. persicae* that correlated with increased toxicity against these insect pests. Cyt2Aa exposed loop3 and loop 4 corresponds to Cyt1Aa loop6 and loop9 respectively (see Supplementary Figure S3). Our data indicated that the Cyt1Aa hybrid toxins constructed in loop6 and loop9 of Cyt1Aa also resulted in improved binding and resulted in proteins that show substantial insecticidal activity against lepidopteran pests. The hybrid mutant constructed in loop 7 of Cyt1Aa also resulted in improved binding and increased toxicity against *M. sexta* and *P. xylostella* larvae. It is evident in Fig. 2B that the Cyt1A-hybrid mutants with increased toxicity against lepidopteran larvae are located in the same face of the Cyt1Aa toxin, suggesting that this side of the toxin is adequate to introduce novel binding sites for the toxin. However, in the case of the Cyt1Aa-hybrid mutants, the seven proteins that were constructed here were not toxic to *A. aegypti* larvae when assayed as crystal-like inclusions or as soluble proteins, in contrast with the reported Cyt2A-hybrid variants that retained mosquitocidal activity when assayed as soluble proteins^22^. In fact the wild type Cyt1Aa was also inactive when tested as soluble protoxin and only showed toxicity as crystal inclusions. We do not know the reasons for this discrepancy. Hybrid-Cyt1Aa proteins were still able to synergize Cry11Aa and showed partial hemolytic activity, indicating that the Cyt1Aa-hybrid mutants have their correct conformation. It was previously shown that insecticidal activity of Cyt1Aa is not necessary to induce synergism of Cry11Aa or Cry4Ba, since Cyt1Aa mutants affected in oligomerization and toxicity to *A. aegypti* still synergize Cry11Aa^28^. Also, Cyt1Aa is not toxic to *Anopheles albimanus* but it is still able to synergize the toxicity of Cry11Aa and Cry4Ba toxins against this mosquito^26^. Mosquito larvae feed by filtering and soluble proteins are poorly retained in their gut, explaining their low toxicity as soluble proteins. Cyt toxins from Bt do not bind to midgut proteins but they interact with non-saturated membrane lipids, such as phosphatidylcholine, phosphatidylethanolamine and sphingomyelin^29^. Once that Cyt toxin bound to lipids, it forms oligomers that insert into the membrane^28^. It was proposed that the two outer layers of α-helices hairpins swing away from the β-sheet structure, and that these β-strands insert into the membrane forming a β-barrel pore^30^. The mechanism of action of Cyt toxins is not completely understood and an alternative model proposed that they may affect the lipid packing resulting in break down of the membrane due to a detergent like action^16,31^. It could be possible that differences in the mechanism of action of Cyt 2Aa and Cyt1Aa toxins could account for the differences in toxicity of the hybrid Cyt2Aa or Cyt1Aa proteins to *A. aegypti*, this remains to be further analyzed.

Here we show, for the first time, that exchange of loops involved in insect specificity among toxins that are not related structurally is feasible and could provide tools for changing insect specificity and potentially for providing tools for countering resistance. A recent publication showed that Cyt1Aa-BinA hybrid toxins can be constructed resulting in hybrid-toxins with a broad-spectrum against mosquitoes including *Anopheles gambiae, C. quinquefasciatus* and *A. aegypti*^32^. These data indicate that Cyt1Aa toxin may accept larger domains and produce active proteins, suggesting that in the future other Cyt1Aa-hybrids could be constructed containing entire domains that could effectively enhance a lethal effect by Cyt1Aa in other targets. Potentiation of the insecticidal activity of these novel hybrid toxins as well as induction of synergism of these hybrid toxins with other lepidopteran specific toxins would be goal for the future work.

## Materials and Methods

### Construction of Cyt1Aa-hybrid toxins

A set of primer pairs was designed to insert the loop 3 amino acid sequence from Cry1Ab toxin (FRSGFSNSSVSI) into *cyt1Aa* gene by a PCR strategy. Two PCR reactions (named PCR1 and PCR2, see Table 2) were performed for each construction. The 5′ end of the reverse primers used in the PCR1 reactions and the forward primers used in the PCR2 reactions, contain the sequence of loop3-*cry1Ab*, while the 3′ end of these primers contains a sequence homologous to each loop region of the target *cyt1Aa* gene (Supplementary Figure S1, Table 2). Primer pairs for PCR1 and PCR2 amplifications were used in two separated PCR reactions with Vent DNA polymerase (NEB, Ipswich, MA) using plasmid pWF45 containing *cyt1Aa* gene^33^ as template, as follows: 3 min at 95 °C, followed by 35 cycles of denaturation at 95 °C for 30 sec, annealing at 55 °C for 30 sec and extension at 72 °C for 35 sec, finally an additional extension at 72 °C for 3 min was performed. After amplification, the two PCR products were purified using the PCR purification kit Qiagen (Qiagen, Hilden, Germany) as described by the manufacturer. The purified PCR products were annealed and used as templates in an additional PCR reaction with the forward primer of PCR1 and the reverse primer of PCR2 to amplify a 400 bp PCR-product that was used as mega-primer in the mutagenesis of *cyt1Aa* gene (Supplementary Figure S1). Conditions for these PCR reactions and purification of the final 400 bp PCR-product containing the loop3 from *cry1Ab* inserted into each loop region of *cyt1Aa* were as described above. Finally, the 400 bp PCR-products were used as mega-primers in the PCR-mutagenic reactions using Phusion polymerase (NEB) and plasmid pWF45 as template as follows: 98 °C for 30 sec followed by 35 cycles of denaturation at 98 °C for 15 sec, annealing at 55 °C for 1 min and extension at 72 °C for 5 min for loop1, loop2, loop4, loop5 and loop 6, or for 7 min for loop3, loop7, loop8, loop9 and loop10. Finally, an additional extension at 72 °C for 7 min was performed. After amplification, the PCR products were purified with PCR purification kit Qiagen (Qiagen) and treated with DpnI enzyme for 2 h at 37 °C to digest the methylated DNA and thus eliminate pWF45 template DNA. The products of these reactions were purified as above and used to transform DH5α *E. coli* strain. Transformant cells were selected on LB plate containing 100 μg/mL ampicillin (Amp). Plasmids were purified using Wizard plus DNA purification system from Promega (Promega, Madison WI). After DNA sequencing at facilities of the Institute of Biotechnology UNAM, the plasmids containing the *cyt1Aa-hybrid* mutant genes with the loop3 from *cry1Ab* were transformed into the acrystalliferous Bt 407^−^ strain as previously described^34^. Transformant were selected in erythromycin (Erm) 10 μg/mL at 30 °C. The mutation in the selected Bt colonies was confirmed after plasmid purification using Wizard plus DNA purification system (Promega) and PCR amplification using these plasmids from Bt as templates with the forward primer of PCR1 and the reverse primer of PCR2 for each insertion (Table 2) and sequencing of PCR products.

**Table 2:** Mutagenic oligonucleotides.

Cyt1Aa Loop	PCR	Oligonucleotide	Sequence
**1**	2	F-loop1asa	TTTAGAAGTGGATTTAGTAATAGTAGTGTAAGTATTTCTATTAACGAAATTGATAATCC
		R-loop1 + 200	TTACATTTTGATCAACATAACTCA
	1	F-loop1 − 200	AATTTATTATGTTACTTTATATTTGAT
		R-loop1asa	ACTATTACTAAATCCACTTCTAAAAAGAAGATTATTGATTTCATTTGG
**2**	2	F-loop2asa	TTTAGAAGTGGATTTAGTAATAGTAGTGTAAGTATTTATATATTGCAAGCAATTATGTTA
		R-loop1 + 200	TTACATTTTGATCAACATAACTCA
	1	F-loop1 − 200	AATTTATTATGTTACTTTATATTTGAT
		R-loop2asa	ACTATTACTAAATCCACTTCTAAAATTCGGATTATCAATTTCGTTAATAGA
**3**	2	F-loop3asa	TTTAGAAGTGGATTTAGTAATAGTAGTGTAAGTATTGATGCCCTACGCTTTAGTAT
		R-loop3 + 200	ACTTAATGCAACTCCTAATACAGT
	1	F-loop3 − 200	ATTAGAAGATATAAAGGTAAA
		R-loop3asa	AATACTTACACTACTATTACTAAAACCAAAATCTGTAGAAGTGG
**4**	2	F-loop4asa	TTTAGAAGTGGATTTAGTAATAGTAGTGTAAGTATTGGTGVTGTAGTGAGTTATGTTGAT
		R-loop3 + 200	ACTTAATGCAACTCCTAATACAGT
	1	F-loop4 − 200	TAAATCCATGGAAAACCCCTCAAT
		R-loop3asa	AATACTTACACTACTATTACTAAACATCGGTGTAATTGTGTTTGCGAT
**5**	2	F-loop5asa	TTTAGAAGTGGATTTAGTAATAGTAGTGTAAGTATTTTAAGTGGATCTGTAATAGATCAA
		R-loop6 + 200	TTACTGCTGATACTTTAATTTCAA
	1	F-loop6 − 200	GATGCCCTACGCTTTAGTATG
		R-loop5asa	ACTATTACTAAATCCACTTCTAAATGCAACTCCTAATACAGTTTTTAACACTTC
**6**	2	F-loop6asa	TTTAGAAGTGGATTTAGTAATAGTAGTGTAAGTATTTTAACTGCAGCAGTTACAAATACG
		R-loop6 + 200	TTACTGCTGATACTTTAATTTCAA
	1	F-loop6 − 200	GATGCCCTACGCTTTAGTATG
		R-loop6asa	ACTATTACTAAATCCACTTCTAAATTGATCTATTACAGATCCACTTAA
**7**	2	F-loop7asa	TTTAGAAGTGGATTTAGTAATAGTAGTGTAAGTATTACTCAAAAAAATGAAGCATGGAT
		R-loop6 + 200	TTACTGCTGATACTTTAATTTCAA
	1	F-loop7 − 200	CCCTACGCTTTAGTATG
		R-loop7asa	ACTATTACTAAATCCACTTCTAAAATTTAAATTTGTAAACGTAT
**8**	2	F-loop8asa	TTTAGAAGTGGATTTAGTAATAGTAGTGTAAGTATTTGGATTTTCTGGGGCAAGGAA
		R-loop6 + 200	TTACTGCTGATACTTTAATTTCAA
	1	F-loop7 − 200	CCCTACGCTTTAGTATG
		R-loop8asa	ACTATTACTAAATCCACTTCTAAATGCTTCATTTTTTTGAGTAT
**9**	2	F-loop9asa	TTTAGAAGTGGATTTAGTAATAGTAGTGTAAGTATTCAAACAAATTACACATACAAT
		R-loop9 + 200	TAATGGTTGTGCAAATTTCAA
	1	F-loop9 − 200	GGTGCTGTAGTGAGTTATGTTGAT
		R-loop9asa	ACTATTACTAAATCCACTTCTAAAATTAGCAGTTTCCTTGCCCCA
**10**	2	F-loop10asa	TTTAGAAGTGGATTTAGTAATAGTAGTGTAAGTATTGGCGTTATGTATTGTGTACC
		R-loop9 + 200	TAATGGTTGTGCAAATTTCAA
	1	F-loop9 − 200	GGTGCTGTAGTGAGTTATGTTGAT
		R-loop10asa	ACTATTACTAAATCCACTTCTAAAACCAGTTTGGGCATTTTGGAT

Underlined is the sequence of loop3 from *cry1Ab* gene.

### Production of Cyt1Aa, Cry11Aa and Cry1Ab wild type and mutant proteins

Cyt1Aa, Cyt1Aa-hybrid, Cry11Aa, Cry1Ab or Cry1Ab-G439D protoxins were produced in *B. thuringiensis* 407^−^acrystalliferous strain transformed with wild type or mutated plasmids. Plasmid pWF45 has *cyt1Aa* gene^32^ or the new constructions of *cyt1Aa-hybrid* genes. Plasmid pCG6 contains *cry11Aa* gene^35^. Plasmids pHT315-Ab and pHT315-AbG439D contain *cry1Ab* gene or *cry1Ab*-G439D mutated gene, respectively^24^. Bt transformant strains were grown for four days at 30 °C in solid nutrient broth sporulation medium^36^ supplemented with 10 μg/mL Erm for Cyt1Aa or 25 μg/mL Erm for Cry11Aa. Spores and crystals were washed three times with 0.3 M NaCl, 0.01 M EDTA, pH 8.0 by centrifugation for 10 min at 10,000 rpm at 4 °C and the pellet stored at −20 °C. The crystal inclusions of Cry11Aa, Cry1Ab and Cry1Ab-G439D were purified by centrifugation in sucrose gradients as described^37^. Crystals of Cry1Ab and Cry1Ab-G439D proteins were solubilized in 50 mM Na_2_CO_3_, 0.2% β-mercaptoethanol, pH 10.5, and activated with trypsin in a mass ratio of 1:20 w/w (1 h, at 37 °C). Phenylmethylsulfonyl-fluoride (1 mM final concentration) was added to stop proteolysis.

The crystals of Cyt1Aa and crystal-like inclusions of Cyt1Aa-hybrid proteins were purified by the aqueous two-phase system as previously described^38^. This aqueous two-phase system is constituted by phosphate buffer 40% (15 gr K_2_HPO_4_, 5 gr KH_2_PO_4_, 30 gr H_2_O) and polyethylene glycol (PEG) 40% (20 gr PEG 4000, 30 gr H_2_O). Each spore/crystal suspension was suspended in 0.1% Triton X-100 (0.2 gr spore/crystal, 0.2 ml 0.1% Triton X-100 v/v), one mL H_2_O and 1.6 gr of PEG at 40% were added and mixed in vortex. One gr of KHPO_4_ at 40% was added, and suspension was mixed again by vortex. The samples were centrifuged 1 min at 500 rpm. The crystals in the interphase were recovered and stored at 4 °C. The Cyt1A proteins were solubilized 1 h at 4 °C in 50 mM NaOH, 1 mM DTT, 1 mM PMSF, agitation at 700 rpm and centrifuged for 10 min at 10,000 rpm 4 °C. The soluble protoxins were recovered in the supernatant. Cyt1Aa protoxin was activated with trypsin 1:50 (Trypsin: Cyt1Aa) ratio (Sigma-Aldrich Co., St Louis, MO) w/w for 1 h at 4 °C and 650 rpm. Protein concentrations were determined by the Bradford assay and the protein profile analyzed in SDS-PAGE with 15% acrylamide.

### Western blot assays

Protein samples were boiled 5 min in Laemmli sample loading buffer, loaded in SDS-PAGE, and electrotransferred onto a PVDF membrane 45 min at 350 mA (Millipore, Bedford, MA). The membrane was blocked with PBS, 0.1% Tween 20, 5% skimmed milk for 1 h with agitation. The membrane was then washed three times with PBST (PBS 0.1% Tween 20). Cyt1Aa protein was detected using the anti-Cyt1Aa polyclonal antibody (1:30000 in PBST, 1 h with agitation) washed three times with PBST and incubated with the secondary antibody coupled with horseradish peroxidase (HRP) (Santa Cruz Biotechnology, Dallas TX) (1:30000 in PBST, 1 h with agitation), washed three times with PBST followed by incubation with luminol Sc-2048 (Santa Cruz Biotechnology) as described by the manufacturers. The membrane was visualized in the Imager 600 (Amersham, Pittsburgh, PA). Molecular mass markers used in all SDS-PAGE were Page ruler plus pre-stained protein ladder (Thermo Scientific, Walthman MA).

### Expression and purification of CAD, APN1 and ALP proteins

CAD and ALP from *M. sexta* larvae were previously cloned end heterologous expressed in *E. coli* cells^17,23^. The CAD fragment (CR12) was expressed in *E. coli* ER2566 strain and ALP was expressed in *E. coli* BL21 (DE3) strain (Invitrogen, Carlsbad CA), both proteins were induced with 1 mM isopropyl β-D-1-thiogalactopyranoside (IPTG) and inclusion bodies were solubilized with 8 M urea solution. The CR12 peptide and ALP protein were purified through a nickel affinity column and eluted with 250 mM imidazole in PBS buffer as previously described^17,23^.

For cloning the APN1 gene, the total RNA from 3rd instar *M. sexta* midguts was extracted using RNeasy Mini kit (Qiagen). APN1 sequence was obtained from GenBank accession number: AF123313. Primers for amplification were APN1-Rev (*Eco*RI) TAC AGA ATT CCA TGC TGC GGG ACC CGA GCT ACC GC and APN1-For (*Hind*III) TCT TAA GCT TGC TAC CAT GTT AAT GGC AAG TGT G. A PCR product of 3 kbp was amplified and digested with *Eco*I and *Hind*III restriction enzymes, cloned into pET22b vector and transformed into *E. coli* BL21 (DE3) cells (Invitrogen). Plasmid was purified and confirmed by DNA sequencing at the facilities of Instituto de Biotecnología-UNAM.

Transformants were grown overnight with agitation at 37 °C in 5 ml of LB broth containing 50 μg/ml Amp. One hundred μl of the overnight culture were used to inoculate 100 ml of 2xTY broth 100 μg/ml ampicillin in a 250 ml flask and incubated until an OD_600_ of 0.7 was obtained. One mM IPTG was added and the culture was incubated for additional 6 h at 30 °C. The culture was centrifuged at 5000 rpm for 15 min at 4 °C and the pellet suspended in STE buffer (10 mM Tris–HCl, 1 mM EDTA, 8 M Urea, pH 8). After sonication (5 min on ice), and centrifugation (70,000 rpm, 30 min at 15 °C), the supernatant was recovered and subjected to affinity purification using Ni-Agarose beads (Qiagen). The column was washed with 35 mM imidazole in PBS buffer, pH 7.5, eluted with 250 mM imidazole and gradually dialyzed against PBS buffer.

### ELISA binding assays

ELISA plates were coated with 0.5 μg of *M. sexta* CR12 CAD fragment corresponding to cadherin repeat 12 (CAD-CR12), APN1, or ALP in 100 μl of PBS per well over night at 4 °C. Plates were washed three times with PBS and blocked with 200 µl/well of PBS-M (PBS, 2% skim milk) for 2 h at 37 °C and washed five times with PBS. Different concentrations of Cry1Ab wild type or Cry1Ab-G439D mutant (6.25 to 100 nM) in a total 100 μl volume of PBST (PBS + 0.1% Tween 20.) for 1 h at 37 °C. The unbound toxins were removed by four washes with PBST. The bound toxins were detected using 100 μl PBST buffer containing rabbit anti-Cry1Ab (1: 10,000) antibody for 1 h at 37 °C. After three washings with PBST, we added 100 μl of PBST containing the anti-rabbit conjugated with HRP antibody for 1 h at 37 °C. Finally, three washes with PBST were performed and each well of the plates were incubated with 100 μl of substrate mixture (6 mg/ml *o*-phenylenediamine (Sigma) and 10 μl H_2_O_2_ in 0.1 M phosphate buffer). Reaction was stopped by adding 50 µl of 6 M HCl and measured at OD 490 nm using an ELISA micro plate reader. Each experiment was performed in duplicate with a total of six repetitions.

Binding of hybrid-Cyt1Aa toxins to CAD, APN1, or ALP was also analyzed. ELISA plates were coated with 0.5 μg of the each receptor as described above and incubated with Cyt1Aa or hybrid-Cyt1Aa (50 nM) in a total 100 μl volume of PBST for 1 h at 37 °C. The unbound toxins were removed as above and the bound toxins were detected using 100 μl PBST buffer containing rabbit anti-Cyt1A (1:4,000) antibody for 1 h at 37 °C. After three washings with PBST, 100 μl of PBST containing the anti-rabbit HRP-conjugated antibody was added and incubated for 1 h at 37 °C. Finally, plates were washed and reveled as described above. Each experiment was performed in duplicate with a total of six repetitions for each hybrid-Cyt1Aa toxin.

### Insect bioassays

*Aedes aegypti, M. sexta* and *P. xylostella* insects were reared at Instituto de Biotecnologia facilities, at 28 °C, 75% humidity, with a 12 h: 12 h, light: dark, photoperiod. Bioassays against *A. aegypti* were performed with different concentrations (100 to 10000 ng/ml) of spore/crystal suspensions of Cyt1Aa or hybrid-Cyt1Aa against 10 early 3^th^-instar larvae in 10 ml of dechlorinated water. Bioassays with soluble protoxin proteins were also performed using the same concentrations as described above in 1 ml water and 10 first-instar larvae. Negative control (dechlorinated water) was included in the bioassay. Larvae viability was examined after 24 and 48 h. The mean lethal concentration (LC_50_) was determined by Probit analysis (Polo-Plus LeOra Software) using statistical parameters using data obtained in triplicate from three independent assays.

Toxicity bioassays against *M. sexta* neonate larvae or *P. xylostella* third instar larvae were performed by the surface contamination method in 24 well plates with artificial diet. The toxin solution was poured on the diet surface and allowed to dry. Larvae were placed on the dried diet surface and the mortality was monitored after 7 and 15 days. Twenty-four larvae were used per toxin concentration in triplicate.

The evaluation of synergism between wild type Cyt1Aa and Cry11Aa proteins was done as previously described^26,39^. The synergism was analyzed using three different protein ratios of Cyt1Aa:Cry11Aa (1:1, 1.3 and 1:5 in a final total protein concentration of 50 ng). The theoretical toxicity of each ratio mixture was evaluated according to Tabashnik’s equation^39^, assuming a simple additive effect. The synergism factor (SF) was calculated by dividing the theoretical toxicity by the observed toxicity of the mixture in bioassays. SF values >1 indicates synergism as previously described^39^.

The synergism among Cyt1Aa-hybrid proteins and Cry11Aa was evaluated as described before^26^. Briefly, we used the formula S_*(ab)*EXP_ = S_(*a*)OBS_ × S_(*b*)OBS_ where S_*(ab)*EXP_ is the proportion of larvae expected to survive to the exposure of a mixture of toxins *a* and *b*, S_(*a*)OBS_ and S_(*b*)OBS_ are the observed proportion of larvae that survived to the exposure to toxin *a* or toxin *b*, respectively. Ten larvae were used per toxin concentration and per mixture of toxins. Mortality was analyzed after 24 h. The expected mortality for larvae that were exposed to the mixture of toxins *a* and *b* was calculated as (1 − S_*(ab)*EXP_) × 100% and the expected numbers of dead and live larvae were calculated by multiplying the expected mortality and survival rates by the sample size used when each toxin was tested separately. These assays were done in triplicate. Finally the Fisher’s exact test was used to determine if a significant difference occurred between observe and expected mortality data.

### Hemolysis assays

These assays were done as previously described^40^. Rabbit red blood cells were washed three times in buffer A (0.1 M dextrose, 0.07 M NaCl, 0.02 M sodium citrate, 0.002 M citrate, pH 7.4) and diluted to 2 × 10^8^ cells/ml in buffer A. Samples containing 20 μl of washed blood cells and Cyt1Aa toxin (20–1200 ng) in a final volume of 200 μl of buffer A were incubated at 37 °C for 30 min in 96 wells microtiter plates. The supernatants were collected in a new microtiter plate after centrifugation at 2,500-x *g* for 5 min at 4 °C and hemolytic activity was quantified by measuring the absorbance of the supernatant at 405 nm. Positive control showing 100% hemolysis was defined after incubation of the same volume of rabbit red blood cells with dechlorinated H_2_O. Negative controls were red blood cells incubated with buffer A. These assays were performed three times in triplicate each time. A *t*-test was performed using the statistical program GraphPad Prism.

## Electronic supplementary material

Supplementary information

## References

[1] Pardo-López L, Soberón M, Bravo A (2013). *Bacillus thuringiensis* insecticidal toxins: Mode of action, insect resistance and consequences for crop protection. FEMS Microbiol. Rev..

[2] James, C. 20th Anniversary (1996 to 2015) of the Global Commercialization of Biotech Crops and Biotech Crop Highlights in 2015. *ISAAA Brief* No. 51. ISAAA: Ithaca, NY (2015).

[3] Soberón M, López-Díaz JA, Bravo A (2013). Cyt toxins produced by *Bacillus thuringiensis*: A protein fold conserved in several pathogenic microorganisms. Peptides..

[4] Porcar M, Grenier A-M, Federici B, Rahbe Y (2009). Effects of *Bacillus thuringiensis* δ-endotoxins on the pea aphid (*Acyrthosiphon pisum*). Appl. Environ. Microbiol..

[5] Pal N, Yamamoto T, King GF, Waine C, Bonning B (2013). Aphicidal efficacy of scorpion and spider derived neurotoxins. Toxicon..

[6] Federici BA, Bauer LS (1998). Cyt1Aa protein of *Bacillus thuringiensis* is toxic to the cottonwood leaf beetle, *Chrysomela scripta*, and suppresses high levels of resistance to Cry3Aa. Appl. Environ. Microbiol..

[7] Stockhoff, B. & Conlan, C. Controling hemipteran insects with *Bacillus thuringiensis* US patent 5723440 (1998).

[8] Wellman-Desbiens E, Cote J-C (2013). 2005 Development of *Bacillus thuringiensis*-based assay on *Lygus hesperus*. J. Econ. Entomol..

[9] van Frankenhuyzen K (2013). Cross-order and cross-phylum activity of *Bacillus thuringiensis* pesticidal proteins. J. Invertebr. Pathol..

[10] van Frankenhuyzen K, Tonon A (2013). Activity of *Bacillus thuringiensis* Cyt1Ba crystal protein against hymenopteran forest pests. J. Invertebr Pathol..

[11] Pérez C (2005). Bti Cry11Aa and Cyt1Aa toxins interactions support the synergism-model that Cyt1Aa functions as membrane-bound receptor. Proc. Natl. Acad. Sci. USA.

[12] Cantón PE, Reyes EZ, RuízdeEscudero I, Bravo A, Soberon M (2011). Binding of *Bacillus thuringiensis* subsp*. israelensis* Cry4Ba to Cyt1Aa has an important role in synergism. Peptides..

[13] Wirth M, Georghiou GP, Federici BA (1997). CytA enables CryIV endotoxins of *Bacillus thuringiensis* to overcome high levels of CryIV resistance in the mosquito *Culex quinquefasciatus*. Proc. Natl. Acad. Sci. USA.

[14] Li J, Koni PA, Ellar DJ (1996). Structure of the mosquitocidal deltaendotoxin CytB from *Bacillus thuringiensis* ssp. *kyushuensis* and implications for membrane pore formation. J. Mol. Biol..

[15] Knowles BH (1989). A cytolytic delta-endotoxin from *Bacillus thuringiensis* var. *israelensis* forms cation-selective channels in planar lipid bilayers. FEBS Lett..

[16] Butko P (2003). Cytolytic toxin Cyt1Aa and its mechanism of membrane damage: data and hypothesis. Appl. Environ. Microbiol..

[17] Pacheco S (2009). Domain II loop 3 of *Bacillus thuringiensis* Cry1Ab toxin is involved in a “ping-pong” binding mechanism with *Manduca sexta* aminopeptidase-N and cadherin receptors. J. Biol. Chem..

[18] Gomez I, Dean DH, Bravo A, Soberon M (2003). Molecular basis for *Bacillus thuringiensis* Cry1Ab toxin specificity: Two structural determinants in the *Manduca sexta* Bt-R1 receptor interact with loops α-8 and 2 in domain II of Cy1Ab toxin. Biochemistry..

[19] Rajamohan F, Alzate O, Cotrill JA, Curtiss A, Dean DH (1996). Protein engineering of *Bacillus thuringiensis* delta-endotoxin: mutations at domain II of CryIAb enhance receptor affinity and toxicity toward gypsy moth larvae. Proc. Natl. Acad. Sci. USA.

[20] Liu XS, Dean DH (2006). Redesigning *Bacillus thuringiensis* Cry1Aa toxin into a mosquito toxin. Prot. Eng. Design. Selec..

[21] Shao E (2016). Loop replacements with gut-binding peptides in Cry1Ab domain II enhanced toxicity against the brown planthopper, *Nilaparvata lugens* (Stal). Scientific Rep..

[22] Chougule NP (2013). Retargeting of the *Bacillus thuringiensis* toxin Cyt2Aa against hemipteran insect pest. Procs. Natl. Acad. Sci. USA.

[23] Flores-Escobar B, Rodríguez-Magadan H, Bravo A, Soberon M, Gomez I (2013). *Manduca sexta* aminopeptidase-N and alkaline phosphatase have a differential role in the mode of action of Cry1Aa, Cry1Ab and Cry1Ac toxins from *Bacillus thuringiensis*. Appl. Environ. Microbiol..

[24] Rodríguez-Almazán C (2009). Dominant negative mutants of *Bacillus thuringiensis* Cry1Ab toxin function as anti-toxins: Demonstration of the role of oligomerization in toxicity. PloS ONE..

[25] Cohen S (2011). Cyt1Aa toxin: crystal structure reveals implications for its membrane-perforating function. J. Mol. Biol..

[26] Fernández-Luna MT (2010). Single-concentration tests show synergism among *Bacillus thuringiensis* subsp. *israelensis* toxins against the malaria vector mosquito *Anopheles albimanus*. J. Invertebr. Pathol..

[27] Canton PE, López-Días JA, Gill SS, Bravo A, Soberon M (2014). Membrane binding and oligomer membrane insertion are necessary but insufficient for *Bacillus thuringiensis* Cyt1Aa toxicity. Peptides..

[28] López-Diaz JA, Cantón PE, Gill SS, Soberón M, Bravo A (2013). Oligomerization is a key step in Cyt1Aa membrane insertion and toxicity but not necessary to synergize Cry11Aa toxicity in *Aedes aegypti* larvae. Environ. Microbiol..

[29] Thomas WE, Ellar DJ (1983). Mechanism of action of *Bacillus thuringiensis* var *israelensis* insecticidal δ-endotoxin. FEBS Lett..

[30] Promdonkoy B, Ellar DJ (2000). Membrane pore architecture of a cytolytic toxin from. Bacillus thuringiensis. Biochem. J..

[31] Manceva SD, Pustay-Carey M, Butko P (2004). Effect of pH and ionic strength on the cytolytic toxin Cyt1A a florescence spectroscopy study. Biochim. Biophys Acta..

[32] Bideshi DK, Park H-W, Hice RH, Wirth MC, Federeci BA (2017). Highly effective broad spectrum chimeric larvicide that targets vector mosquitoes using a lipophilic protein. Sci. Rep..

[33] Wu D, Johnson JJ, Federici BA (1994). Synergism of mosquitocidal toxicity between CytA and CryIV proteins using inclusions produced from cloned genes of *Bacillus thuringiensis* subsp. *israelensis*. Mol. Microbiol..

[34] Arantes O, Lereclus D (1991). Construction of cloning vectors for *Bacillus thuringiensis*. Gene..

[35] Chang C, Yu YM, Dai SM, Law SK, Gill SS (1993). High-level *cryIVD* and *cytA* gene expression in *Bacillus thuringiensis* does not require the 20-kilodalton protein, and the coexpressed gene products are synergistic in their toxicity to mosquitoes. Appl. Environ. Microbiol..

[36] Schaeffer P, Millet J, Aubert J-P (1965). Catabolic repression of bacterial sporulation. Proc. Natl. Acad. Sci. USA.

[37] Thomas WE, Ellar DJ (1983). *Bacillus thuringiensis* var israelensis crystal deltaendotoxin: effects on insect and mammalian cells *in vitro* and *in vivo*. J. Cell Sci..

[38] Güereca L, Bravo A, Quintero R (1994). Design of an aqueous two-phase system for the purification of ICP from *Bacillus thuringiensis*. Process Biochem..

[39] Tabashnik BE (1992). Evaluation of synergism among *Bacillus thuringiensis* toxins. Appl. Environ. Microbiol..

[40] Rodriguez-Almazán C (2011). The amino- and carboxyl-terminal fragments of the *Bacillus thuringiensis* Cyt1Aa toxin have differential roles on toxin oligomerization and pore formation. Biochemistry..

