# Supplementary material for: Engineering Bacillus thuringiensis Cyt1Aa toxin specificity from dipteran to lepidopteran toxicity
Source: Sci Rep. 2018 Mar 21;8:4989. doi: 10.1038/s41598-018-22740-9 (PMC5862903; doi:10.1038/s41598-018-22740-9)

Supplementary information

Engineering *Bacillus thuringiensis* Cyt1Aa toxin specificity from dipteran to lepidopteran toxicity

Mary-Carmen Torres-Quintero, Isabel Gómez, Sabino Pacheco, Jorge Sánchez, Humberto Flores, Joel Osuna, Gretel Mendoza, Mario Soberón, Alejandra Bravo*.

Figure S1. Strategy used for construction of hybrid-Cyt1Aa variants

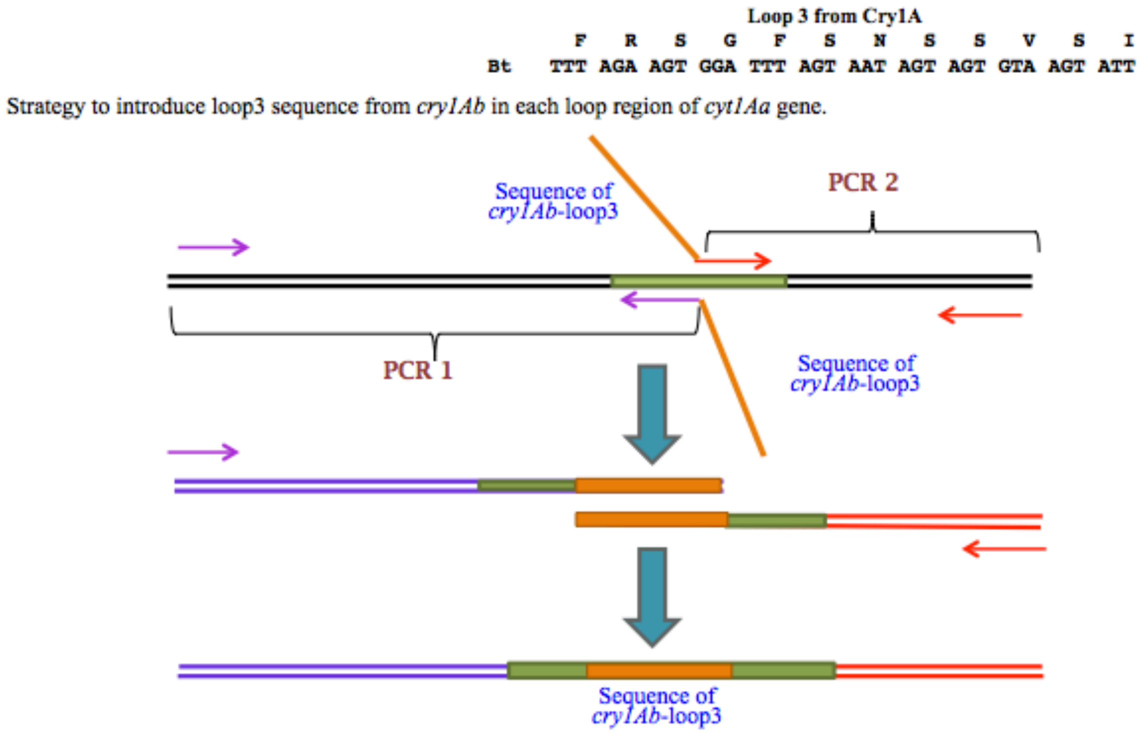

Figure S2. Effect of Cyt1Aa or hybrid-Cyt1Aa variants on *Manduca sexta* larvae. Analysis after seven days of treatment with the Cyt1Aa toxins showing that the larvae treated with hybrid-Cyt1Aa variants (CytL3-loop6, CytL3-loop7 or CytL3-loop9) had a pale color and showed reduced size in comparison with the water control.

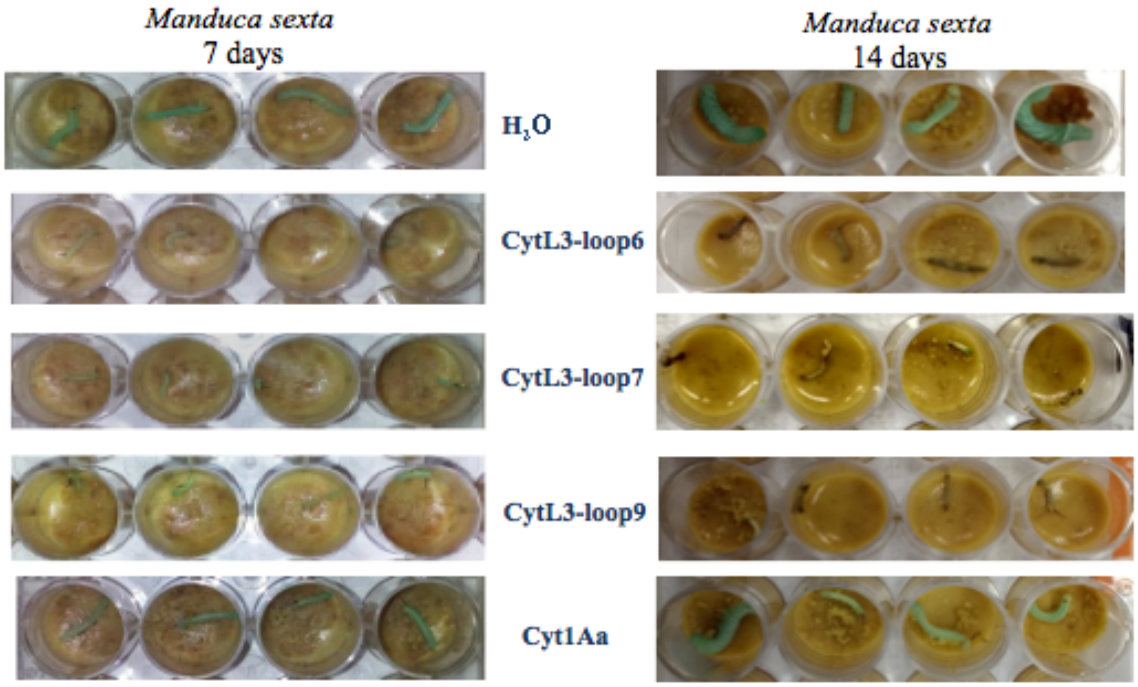

Figure S3. Superposition of three dimensional structures of Cyt1Aa and Cyt2Aa showing the selected loops for insertion of the different peptides.

Ribbons in pink color corresponds to Cyt2Aa structure (1CBY) and ribbons in light blue color corresponds to Cyt1Aa (3RON).

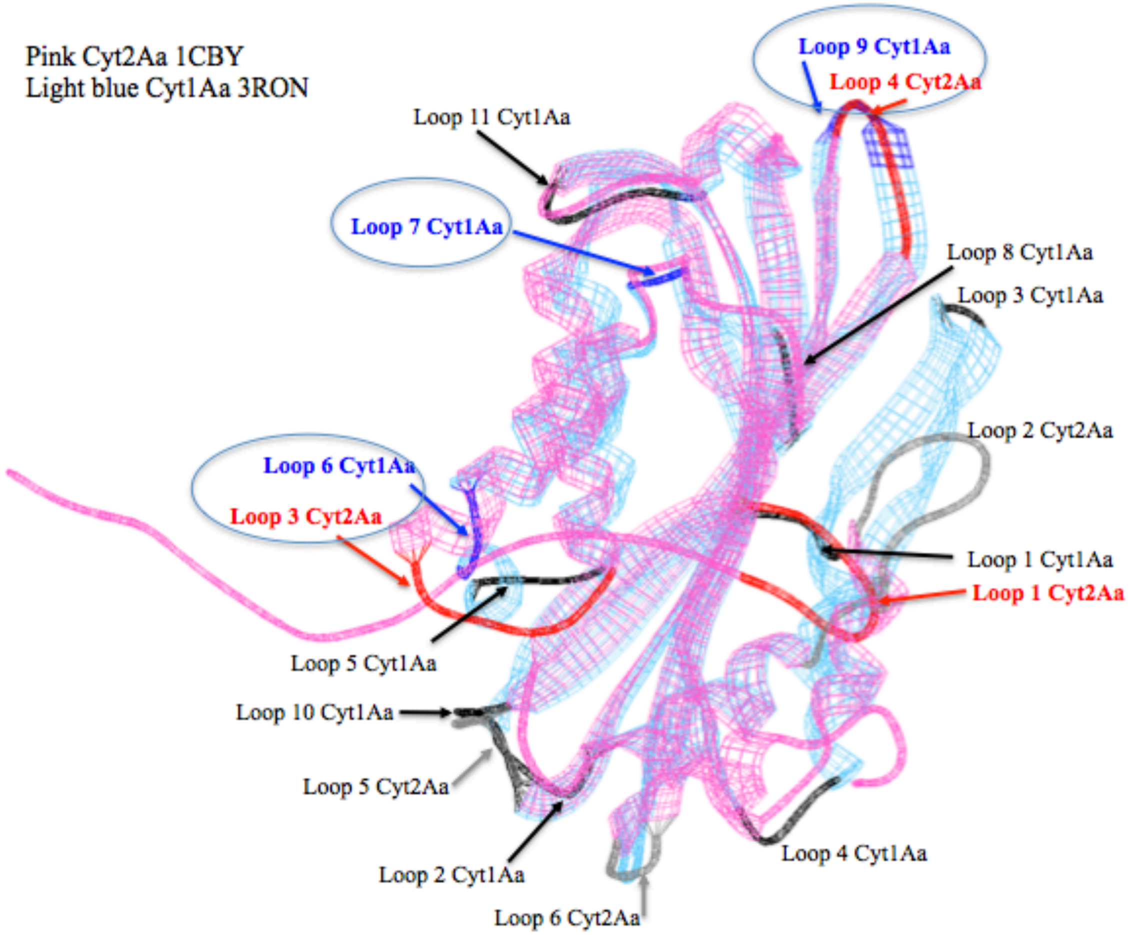

Supplement: Supplementary file 1 — Supplementary information [file 41598_2018_22740_MOESM1_ESM.pdf]
